# A farewell to professor Stefan Agewall from the ESC Working Group on Cardiovascular Pharmacotherapy

**DOI:** 10.1093/ehjcvp/pvag004

**Published:** 2026-02-27

**Authors:** Dobromir Dobrev, Juan-Carlos Kaski, Bianca Rocca, Heinz Drexel, Basil S Lewis, Alexander Niessner

**Affiliations:** Institute of Pharmacology, Faculty of Medicine, University Duisburg-Essen, Essen 45141, Germany; Cardiology Clinical Academic Group, St. George's University Hospitals NHS Trust, London SW17 0QT, UK; Royal Brompton Hospital, London SW3 6NP, UK; Department of Medicine and Surgery, LUM University, S.S. 100 Km. 18, Casamassima 70010, Italy; Vorarlberg Institute for Vascular Investigation and Treatment (VIVIT), Feldkirch 6800, Austria; Private University in the Principality of Liechtenstein, Triesen 9495, Liechtenstein; Vorarlberger Landeskrankenhausbetriebsgesellschaft, Feldkirch 6800, Austria; Drexel University College of Medicine, Philadelphia, PA 19104, USA; Lady Davis Carmel Medical Center and the Technion-Israel Institute of Technology, Haifa 3436212, Israel; Department of Cardiology, Clinic Landstrasse, Vienna Healthcare Group, Vienna 1030, Austria; Division of Cardiology, Department of Internal Medicine II, Medical University of Vienna, Vienna 1030, Austria

Professor Stefan Agewall will step down as Editor-in-Chief of the *European Heart Journal – Cardiovascular Pharmacotherapy* (EHJ-CVP) after serving in the role since 2014. On behalf of the current and former Chairpersons and Nucleus Members of the ESC Working Group on Cardiovascular Pharmacotherapy, we acknowledge his service and contributions to the journal.

EHJ-CVP was established in 2015 as the official peer-reviewed journal of the ESC and the ESC Working Group on Cardiovascular Pharmacotherapy. The EHJ-CVP was established to serve as the official ‘*peer-reviewed journal of the ESC and the ESC WG ESC on Cardiovascular Pharmacotherapy, with a specific focus on clinical cardiovascular pharmacology*’ (https://academic.oup.com/ehjcvp/pages/About). The journal aimed to provide a platform for research at the interface of cardiovascular medicine and pharmacotherapy. Over the past twelve years, Professor Stefan Agewall has defined the journal's scope, editorial processes, and operational structure.

Key milestones during his tenure include the indexing only 2 years after the journal was established, receiving an Impact Factor in 2019, and the transition to full Open Access in January 2025. These developments occurred alongside broader changes and challenges in scientific publishing, including evolving dissemination practices, AI use, increased emphasis on research integrity, and the adoption of new editorial technologies. The journal maintained its continuity and consistency in editorial standards throughout these changes.

Professor Stefan Agewall's editorship emphasized methodological quality, clinical relevance, and structured peer review. The journal has continued to serve both clinical and research communities in cardiovascular pharmacotherapy by engaging authors, reviewers, and editorial colleagues.

At the conclusion of his term, EHJ-CVP remains an established journal with a clearly defined profile and operational framework, with the latest IF of 6.1. The ESC Working Group on Cardiovascular Pharmacotherapy thanks Professor Stefan Agewall for his service and contributions and wishes him success in his future academic and professional endeavours.

## Data Availability

No data have been used for this editorial.

